# Improving Health for People Living With Heart Failure: Focus Group Study of Preconditions for Co-Production of Health and Care

**DOI:** 10.2196/27125

**Published:** 2021-05-11

**Authors:** Anne-Marie Suutari, Johan Thor, Annika M M Nordin, Sofia Kjellström, Kristina Areskoug Josefsson

**Affiliations:** 1 Jönköping Academy for Improvement of Health and Welfare School of Health and Welfare Jönköping University Jönköping Sweden; 2 Department of Internal Medicine and Geriatrics The Highland Hospital Eksjö Sweden; 3 Faculty of Health Studies VID Specialized University Oslo Norway; 4 Department of Behavioral Science Oslo Metropolitan University Oslo Norway

**Keywords:** co-production of health and care, capability, motivation, opportunity, Capability, Opportunity, and Motivation Behavior model, focus groups, heart failure, Sweden, primary care, cardiology, co-production

## Abstract

**Background:**

Co-production of health and care involving patients, families of patients, and professionals in care processes can create joint learning about how to meet patients’ needs. Although barriers and facilitators to co-production have been examined previously in various health care contexts, the preconditions in Swedish chronic cardiac care contexts are yet to be explored. This study is set in the health system of the Swedish region of Jönköping County and is part of system-wide efforts to promote better health for persons with heart failure (HF).

**Objective:**

The objective of this study was to test the usefulness of the Capability, Opportunity, and Motivation Behavior (COM-B) model when assessing the barriers to and facilitators of co-production of health and care perceived by patients with HF, family members of patients with HF, and professionals in a Swedish chronic cardiac care context as a guide for subsequent initiatives.

**Methods:**

Data collection involved 1 focus group interview (FGI) with patients with HF (n=5), 1 FGI with family members of patients with HF (n=5), 1 FGI with professionals in primary care (n=7), and 1 FGI with professionals in cardiac care (n=4). In addition, patients with HF kept diaries of their thoughts regarding co-production. Using a deductive approach to content analysis, underpinned by the COM-B model, barriers and facilitators were categorized into capabilities, opportunities, and motivations to co-produce health and care.

**Results:**

The participants showed limited understanding of co-production as a practice. They appeared to view it as a privilege to be offered to patients on top of traditional care and rarely as an approach for improving health care processes. The interviews revealed the limited health literacy among patients and the struggle of professionals to convey health information to these patients. Co-production was considered to be more resource-intensive than traditional care. Different expectations of stakeholders’ roles were revealed: professionals expected older patients not to want to co-produce health and care, and all participants expected professionals to be in charge of health care services. The family members’ position involved trying to balance their desire to support their relatives with understanding when, how, and with whom to co-produce. Presumed benefits motivated stakeholders: co-production was recognized to motivate patients to improve self-care. However, the participants recognized that motivation to get involved in health and care decisions varies over time among stakeholders.

**Conclusions:**

Co-production can be facilitated by the stakeholders’ motivation. However, varying levels of understanding of co-production, patients’ limited health literacy, unease with power sharing between patients and professionals, and resource constraints are barriers that need to be managed to promote co-produced care and better health for persons living with HF. Further research is warranted to explore how to co-produce health care services with patients with HF and how leaders can facilitate the inevitable cultural change it requires and represents.

## Introduction

### Background

Promotion of the best possible health for persons living with chronic disease is challenging for health systems worldwide. There is growing interest in co-production of health care services—involving patients, families of patients, and professionals in care processes—to create joint learning about how to meet patients’ needs, thus promoting the best possible health [1-6]. Although barriers and facilitators to co-production have been examined previously in various health care contexts [1,2,7-19], the preconditions in Swedish chronic cardiac care contexts are yet to be explored. The present study is set in the health system of the Swedish region of Jönköping County and is part of system-wide efforts to promote better health for persons with heart failure (HF).

### Co-Production of Health and Care

Despite many attempts to define co-production of health care, there is no consensus [2,6,8,20-26]. Loeffler et al [21] proposed that the concept implies a collaboration between patients, families, and professionals at many stages of health care processes: co-planning, co-design, co-delivery, and co-assessment. Osborne et al [6] highlighted the importance of learning about how to co-produce effectively and how to apply the lessons for service improvement, indicating co-learning. In this paper, co-production of health and care is understood to be when patients, family members, and professionals collaborate with shared power in health care processes.

The goal for co-production is best possible health and care [1-6]. Understanding barriers to and facilitators of co-production, previously examined in diverse health care contexts, can in turn guide subsequent improvement initiatives. Patient-related barriers include acute illness [2,9], frailty and old age [9,15], limited health literacy (HL) [9-11], and low self-efficacy and engagement [9,11]. In addition, a reluctance of some professionals to use new ways of working is a barrier to co-production [2,14]. Insufficient communication skills among professionals can also hinder co-production [9-11]. Further barriers are resource constraints, such as staff shortages, poor continuity, and shorter appointments [1,7,9,12,14]. Conditions that can facilitate co-production include individual motivation [13], support from leaders [15-19], and “learning networks” that include patients, nonformal caregivers, and professionals [2].

### Co-Producing Health and Care With Persons Living With HF

HF is a common chronic heart disease that affects 10% of people over the age of 70 years [27]. HF occurs when the heart muscle becomes unable to pump enough blood to meet the body’s needs for blood and oxygen. This causes dyspnea (difficult or labored breathing) and edema (abnormal fluid accumulation in the body) with weight gain and worsened quality of life. Patients with HF usually present with several comorbidities, which add to the burden of disease [27]. Patients with HF also present with cognitive impairment, dementia, and low levels of HL [28-31]. This, in turn, reduces the person’s self-care abilities, with increased use of emergency care, frequent hospitalizations, and a higher mortality rate [32-37].

Clinical guidelines, which promote evidence-based care for HF aimed at reducing mortality and improving quality of life, support a multidisciplinary team approach to HF care including optimal medical and device management, patient involvement in symptom monitoring, and adequate patient education [27]. Self-care skills to include in patient education are understanding HF symptoms, monitoring and recognizing symptoms and signs, and knowing when and how to self-manage diuretic therapy and fluid intake [27,38]. Behaviors of professionals to optimize learning by patients with HF and facilitate shared decision making include recognizing HF disease barriers to communication and providing individualized information [27]. This approach aligns with the idea of co-production, with patients, family members, and professionals collaborating with shared power in health care processes aiming for best possible care.

When preparing co-production initiatives for and with patients with HF in a Swedish cardiac care context, we found a lack of knowledge about barriers and facilitators in this context. To inform future improvement efforts with and for persons with HF, we sought to better understand the contextual conditions and their implications for co-production of health and care. Our exploration of barriers to and facilitators of behavior change in line with co-production was underpinned by a theoretical model of intentional behavior: the Capability, Opportunity, and Motivation Behavior (COM-B) model [39,40].

### Objective

The objective of this study was to test the usefulness of the COM-B model when assessing barriers to and facilitators of co-production of health and care perceived by patients with HF, family members of patients with HF, and professionals in a Swedish chronic cardiac care context, as a guide for subsequent initiatives.

## Methods

### Study Design

This was an explorative qualitative study involving 4 stakeholder groups: (1) patients with HF, (2) family members of patients with HF, (3) professionals working in specialized cardiac care, and (4) professionals working in primary care. Data collection involved focus group interviews (FGIs) [41,42] and participating patients’ health diaries [43,44] kept specifically for this study. The study was vetted and approved by the Swedish Ethical Review Authority (Dnr: 2019-03530). All quotes presented in this paper were anonymized and cannot be traced to specific individuals participating in this study.

### Study Context

The study was conducted in the Highland health district in Region Jönköping County (RJC), Sweden. Primary care centers (PCCs) serve the 115,000 inhabitants in the area. Heart disease, including HF, accounts for a major part of the disease burden in the district’s aging population. Patients with heart disease receive care mostly in PCCs, with access to emergency care and specialized cardiac care in the Highland district hospital as needed. To enable the best possible health and care for these patients, health professionals in primary care and specialty care are expected to cooperate with each other to meet the needs of patients and their family members.

In recent years, RJC—with its long history of systematic improvement efforts [45-48]—has launched several projects involving different stakeholders in society. The “Tillsammans” (“Together”) initiative aims to improve the lives and health of all residents and to shift more care services closer to them, from hospitals to PCCs [49]. The promotion of co-production of health and care is central to this initiative. Although the concept of “co-production of health and care” has lately become more familiar to professionals in RJC, few departments and PCCs have fully adopted the concept.

### Recruitment and Participants

A nurse who was working with patients with HF in primary care but was not a member of the research team suggested eligible patients with HF and their family members for inclusion in this study. Individuals were excluded for the following reasons: (1) under age 18 years; (2) unable to consent to study participation due to acute illness, cognitive impairment, or lack of proficiency in the Swedish language; or (3) had received care from the main author (a practicing cardiologist in the study context). Professionals working in a PCC or in a cardiac ward in RJC were invited by the lead researcher (A-MS) to join the study during workplace meetings and through information letters sent via email. None of the other researchers (JT, AMMN, SK, or KAJ) had a care or working relationship with study participants. The participant characteristics are shown in Table 1.

**Table 1:** Participant characteristics.

Study participants		Gender	Age (years)	Profession
**Patients (n=5)**				
	#1	Male	74	N/A^a^
	#2	Male	66	N/A
	#3	Male	70	N/A
	#4	Female	81	N/A
	#5	Female	76	N/A
**Family members (n=5)**				
	#1	Male	85	N/A
	#2	Female	83	N/A
	#3	Female	89	N/A
	#4	Female	67	N/A
	#5	Female	45	N/A
**Primary care professionals (n=7)**				
	#1	Male	43	Physician
	#2	Female	23	Physiotherapist
	#3	Female	49	Nurse
	#4	Female	46	Nurse
	#5	Female	54	Nurse
	#6	Female	31	Physician
	#7	Female	44	Physician
**Cardiac care professionals (n=4)**				
	#1	Male	38	Physician
	#2	Male	37	Physiotherapist
	#3	Female	50	Nurse
	#4	Female	57	Nurse

^a^N/A: not available.

### Data Collection

Between November 2019 and January 2020, the lead researcher (A-MS) conducted 4 separate FGIs in Swedish with patients with HF, family members of patients with HF, professionals in primary care, and professionals in cardiac care. All participants provided informed written consent prior to data collection. The focus groups were guided by 3 semistructured interview guides developed by the authors, which addressed stakeholders’ perspectives on capabilities, opportunities, and motivations to co-produce health and care (ie, the behavior of interest, all according to the COM-B model). The guides were pilot tested with 2 patients with HF and 2 professionals and revised accordingly prior to the interviews: the word “co-production” was deemed unclear and replaced by “cooperation.” This reflects the difficulty in translating the word “co-production” into Swedish, both semantically and as a previously unfamiliar phenomenon.

The FGIs started with a general question about the participants’ experiences of living with HF or experiences of caring for these individuals. The participants were then encouraged to share their experiences and perspectives of cooperation in health care, assisted by the interview guide. During the FGIs, the interviewer and lead researcher (A-MS) explained co-production of health and care to be when patients, family members, and professionals collaborate with shared power to improve health and care. All interviews ended with participants being given the opportunity to add anything of relevance regarding cooperation in health care that had not been covered during the interviews.

Each FGI lasted for approximately 1 hour. The interviews took place in a conference room in a PCC or the hospital. All interviews were audio-recorded, transcribed verbatim, and deidentified by a clinical documentation assistant with a duty of confidentiality or by the lead researcher.

In addition to the interview, participants living with HF were asked to write diary entries as often as they wanted during a 2-week period about their experiences and thoughts regarding the co-production of health and care to add data to the study. Data collection through patient diaries has previously been shown to be useful for involving patients in health care development [43,44]. All 5 participants living with HF agreed to write a diary entry at home following the FGI and mailed it to the lead researcher.

### Data Analysis

First, the lead researcher (A-MS) performed a qualitative latent content analysis according to Elo and Kyngäs [50]. The analysis included reading through the interview transcripts and patient diaries several times to become familiar with, and make sense of, the data. This was followed by open coding of the material, writing notes and headings in the text while reading it. These notes and headings were transferred to coding sheets and categorized into 2 categories: (1) barriers to and (2) facilitators of co-production of health and care.

Using a deductive approach, these barriers and facilitators were then coded into a categorization matrix developed from the COM-B model (Table 2). The COM-B model highlights conditions in 3 domains that affect behavior change: capability (physical and psychological), opportunity (physical and social), and motivation (reflective and automatic) [39]. Physical capability refers to physical ability, strength, and skills, whereas psychological capability refers to comprehension, knowledge, capacity to engage in the necessary thought processes, memory, and cognition [9,39,51]. Physical opportunity refers to the opportunity afforded by the environment (eg, time, facilities, resources, and availability), whereas social opportunity refers to the opportunity afforded by the milieu (eg, cultural norms and roles, interpersonal influences, and inequalities) [9,39,51]. Reflective motivation refers to intentions, plans, convictions, and considerations, whereas automatic motivation refers to wishes, needs, feelings, and habits [39,51]. This model has previously proven useful in efforts to identify barriers and facilitators to co-production of health and care [9,14] and other co-production–related behaviors such as patient participation in health care safety promotion [52], patients planning advanced care [53], and shared decision making [54].

The quotes chosen to illustrate the results were translated from Swedish into English. The first draft of the analysis was discussed in depth with one of the researchers (KAJ), then further revised before being discussed among all researchers (A-MS, JT, AMMN, SK, and KAJ) until a consensus was reached.

**Table 2:** Barriers and facilitators to co-production of health and care.

COM-B^a^ model domain and condition			Barriers		Facilitators
**Capability**					
	Physical	Impaired physical strength (ie, dyspnea or fatigue)		Sufficient physical strength to engage in co-production	
	Psychological	Lack of knowledge about co-production Impaired mental health Insufficient coping strategies Difficulties understanding health information Difficulties applying health information Difficulties among professionals in handling individuals with poor health literacy Inadequate communication skills		Capability to understand health information Adequate communication skills Existing working practices that promote co-production Capability to adapt to new work methods Support from family members	
**Opportunity**					
	Physical	Fragmented health care system Insufficient leadership support Time and resource constraints		Accessible health care support	
	Social	Expectations of the patients’ role Expectations of the professionals’ role Family members’ role variations			
**Motivation**					
	Reflective	Belief that co-production is unachievable		Belief that co-production improves care Belief that co-production leads to efficient use of resources Plans for how to co-produce	
	Automatic	Reluctance to co-produce		Desire to co-produce	

^a^COM-B: Capability, Opportunity, and Motivation Behavior.

## Results

The results from the qualitative analysis are summarized in Table 2 and described in detail below.

### Capability to Co-Produce Health and Care

#### Physical Capabilities

Some patients with HF reported having the physical capability to co-plan and co-evaluate their own health and care. However, all participants acknowledged varying and sometimes impaired physical health as a real-life challenge for many patients:

You want to live as usual but can’t do anything; you have no energy and are constantly tired.

Diary entry, Patient #4

This was understood to influence the patients’ physical capability to participate in co-production of their own health and care.

#### Psychological Capabilities

##### Understanding of “Co-Production of Health Care”

Patients and family members assumed that knowledge about the organization was necessary to be able to design health care processes and that their lived experiences would not be useful when designing care:

I mean, if you don’t have the knowledge you can come up with any kind of claim, which there is no value in […] To participate in designing the health care services […] My views obviously wouldn’t be worth anything.

FGI with patients

This opinion, which exposed a gap in patients’ sense of capability regarding how to use their lived experiences in health care design, mirrored the professionals’ understanding of co-production. The professionals agreed that patients and family members needed more organizational and medical knowledge to participate in co-production of health care. The professionals’ understanding of co-production included a fear of patients taking charge of medical decisions, and ultimately co-production was understood as a service to be offered to patients on top of traditional health care rather than as an approach to operating and improving health care:

I mean, before we can say that we can implement having patients involved in making decisions concerning their own care, then we must have a good concept to deliver to them and we are not there yet.

FGI with professionals in cardiac care

##### Understanding and Applying Health Information

Poor capability to understand health information was recognized as a major barrier for co-production with patients with HF:

Then, when it comes to information sharing with this group of patients, one notices quite clearly that … I mean if you have pretty severe heart failure, then you don’t have that ability to take in this information and, therefore, it is even more important, at virtually every encounter, to repeat, or to add some new information.

FGI with professionals in cardiac care

Professionals also recognized many patients’ poor capability to apply health information. One example was the fear and difficulty in following instructions of patients with HF related to taking extra diuretics when HF symptoms got worse. While recognizing many patients’ difficulties in understanding and applying health information, the professionals still struggled to get the information across to their patients. Rather than changing strategies to connect with the patients, a common approach seemed to be simply to provide even more information:

The patients don’t have enough knowledge or understanding of what we want to convey to them. Of course, we have to convey more and more, and we have to get them on the track.

FGI with professionals in primary care

The participants recognized that support from family members was important for patients with low levels of HL. Hence, the lack of family member support was identified as a challenge to effective co-production among them:

The problem is that the family members are often not present at health care visits [...]

FGI with professionals in cardiac care

##### Working Practices

Not being invited to co-produce health and care was acknowledged as hindering co-production. Family members, in particular, described how they were often overlooked as a natural part of the clinical microsystem (ie, the frontline place where patients, families, and care teams meet and cooperate in health care) [55]:

But I guess I feel that when you as a family member seek contact [with the health care services], then it has gone quite far [...] then it is quite well thought through and then, when you get turned down or receive a cool response, it gets really tough.

FGI with family members

Professionals noted that they could invite patients and family members to co-produce health and care more frequently:

I think that I could be better at inviting the patient and their family members to participate in the health care services. In other words, to ask questions—“What could you do on your own?” or “What do you think about the treatment?” or something like that. “What would suit you?”

FGI with professionals in primary care

Professionals anticipated growing expectations and willingness to adopt new working procedures:

But, over time, I think that more people [patients] will question [things] and want to participate. […] And I think that this [shift] might apply to professionals as well. We also change.

FGI with professionals in primary care

##### Communication

The participants recognized that communication skills were a key capability for co-production. Patients’ ability to communicate with professionals will typically be impaired when they suffer from acute illness. Professionals discussed how patients’ willingness and capability to communicate with professionals could vary across generations:

But the older person[s], they just assume that the doctor is right. So, they don’t question [things].

FGI with professionals in primary care

Professionals talked about their own capability to listen to patients and notice their needs, rather than to educate and inform them through one-way communication. Patients declared that not being listened to by professionals could potentially lead to hesitation in seeking care:

They [the physicians] just don’t listen. They merely look over your head. […] You feel like you’re in their way and therefore, one simply hesitates to call here even when feeling bad.

FGI with patients

### Opportunity to Co-Produce Health and Care

#### Physical Opportunities

The participants in all 4 focus groups considered the overall organization of the health care systems to be important for successful co-production. Patients and professionals noted that when care is provided by multiple stakeholders in parallel, it is difficult for patients and professionals to know when and which stakeholder to co-produce health and care with:

It is difficult with doctors. In my case, I had a stroke […] If I have trouble with something that has to do with the stroke, then I will call that doctor, and then there are two other doctors who take care of the heart. […] It’s a real mess, I think. You do not know who to turn to.

FGI with patients

Furthermore, they suggested that good continuity of care could facilitate care and support.

All participants expected co-production to be more time- and resource-intensive than traditional care. One physician presumed that insufficient time for health care visits would reduce professionals’ willingness to ask questions and listen to, and consider, the patients’ answers and needs. Patients agreed:

I think that they [the health professionals] would be happy to know more about how we feel, but the question, obviously, is if they have the time to sit and listen to us?

FGI with patients

One nurse expressed doubts regarding the organization’s resources to individualize care:

They [the patients] may need even more support and then the question is can we give—can we tailor it—as much as we would need to?

FGI with professionals in cardiac care

#### Social Opportunities

The FGIs revealed expectations on the roles of patients, family members, and professionals. These role expectations, reflective of a somewhat traditional (“doctor knows best”) health care system, challenge a shift toward patients being treated as equal partners in health care.

##### Patients’ Role

Some professionals said that they thought older persons would have difficulties co-producing health and care. They assumed that older patients were familiar with, and expected, physicians to be in charge. Some professionals also considered patients to be just passive recipients of health information. Still, the professionals expected the patients to take the initiative to acquire more health education:

The patients have to communicate that they want more information and an opportunity to ask questions […] If this kind of initiative doesn’t come from the patients it is very difficult to justify why we should come up with this.

FGI with professionals in cardiac care

Some patients, reflecting a feeling of inferiority in their role relative to that of professionals, imagined that professionals would not appreciate their opinions about organizational and medical matters:

You do not want to be bothersome.

FGI with patients

Also, patients who experienced health care resource constraints felt an obligation to let others with supposedly greater needs get priority.

##### Professionals’ Role

An expectation of the professionals, expressed by both professionals and patients, was that they should be in charge of health care processes. This uneven power balance between patients and clinicians could be caused by old traditions but also by professionals’ discomfort over allowing patients to have more influence over their own health and care:

It is known that HF patients want to take more responsibility for their own illness, that they want to be involved, and it may be we who think that this is our responsibility and may not dare to hand it over to the patient.

FGI with professionals in primary care

##### Family Members’ Role

Family members expressed much worry for their sick relatives while wishing to support them. However, they also expressed uncertainty over when and how to co-produce health and care:

Sometimes you don’t know when to intervene [...] and then you think that then, they [the health care professionals] are surely on top of it all.

FGI with family members

Not all patients expected family members to be involved in their health care:

Well, it’s not something one counts on, to be able to get help [from family members] every day.

FGI with patients

Uncertainty over expectations as well as over when and how to co-produce health and care put family members in a difficult position.

### Motivation to Co-Produce Health and Care

#### Reflective and Automatic Motivations

The participants predominantly spoke about co-producing in one-on-one interactions. Only 1 primary care nurse mentioned having experienced patient participation in health care process co-design efforts. Although there was motivation to co-produce, participants noted that some patients might not want to be actively involved in their own care:

Some [patients] want to do a lot and be heavily involved and become great experts on their own illness, while others become more passive and just say: “You decide.”

FGI with professionals in primary care

This calls for organizational flexibility, which may be difficult to get to work in practice. Some participants even imagined that co-production was unachievable:

Of course, it’s a dream scenario that [a] patient, family members and health care professionals work together but I don’t really believe in that possibility!

Diary entry, Patient #2

Several participants pointed to co-production benefits. On the frontline level, participants talked about how cooperation promoted patients’ and family members’ sense of security in everyday life and improved the quality of health and care. One patient acknowledged that co-production could promote professionals’ learning about living with the disease, thus improving the capability to design appropriate care:

They [the physicians] read a lot about it when they educate themselves, but this does not say anything about how we feel and how we experience it [living with disease]. It varies. So, it must be nice for a doctor to find out how we feel, to be able to do the right things.

FGI with patients

Professionals thought that co-production could potentially encourage patients to perform better self-care supported by improved team communication and a patient-centered approach focused on the patients’ needs.

All participants assumed that health care visits focusing on cooperation to meet patients’ care needs would be more time-consuming (than the usual care provided today) but still worthwhile in the long run for both patients and organizations:

It takes time there and then, but in the long run there will probably be fewer care visits.

FGI with professionals in primary care

Professionals assumed a reduction in unplanned care utilization:

I can imagine that if they [patients] feel involved and can cooperate there will perhaps be fewer readmissions.

FGI with professionals in cardiac care

## Discussion

### Principal Findings

Most barriers to co-production of health and care concerned the domain of capability, including difficulty understanding the term “co-production.” Participants had limited understanding of the concept as a practice and appeared to view it as a privilege to be offered to patients on top of traditional care and rarely as an approach for improving health care processes. The FGIs revealed poor HL and low self-efficacy in co-producing among patients. Professionals’ struggle to convey useful information to patients could be viewed as an indication of insufficient organizational HL. Communication skills and the inclusion of stakeholders in co-production emerged as key facilitating capabilities.

In terms of physical opportunities, co-production was considered to demand more time and resources than traditional care. Regarding social opportunities, different role expectations of patients, family members, and professionals were revealed. Professionals expected older patients in particular not to want to be involved in their own health and care. Both professionals and patients expected the professionals to be in charge of and responsible for health care services. The data revealed that family members are in a difficult position when balancing their desire to support their sick relative with uncertainty of when, how, and with whom to co-produce health and care.

In terms of motivation, presumed benefits were identified. It was recognized that co-production of health meant working with a patient-centered approach that promoted patients’ and family members’ sense of security in everyday life and motivated patients to improve their self-care. This was believed to improve the quality of health and care and, ultimately, to reduce unplanned care in favor of more planned care. However, the participants recognized that motivations to co-produce health and care vary over time among patients and professionals.

### Comparison With Prior Work

In this study, respondents believed that co-production could improve the quality of health and care by considering patients’ experiences when designing health care services and promoting patients’ self-care abilities. These beliefs mirror those found in previous studies [1-5]. Vennik et al [1] suggested that co-production facilitates health care process improvement through the use of patients’ experiences. Elwyn et al [3] proposed that co-production of health and care empowers people to cope with disease through the promotion of patient resilience and autonomy. However, the professionals recognized that not all patients and family members want to participate in health and care decisions, highlighting patient and family member diversity regarding capabilities and health status as challenges to standardization of co-production [2]. Virlée et al [11] suggested that patients’ motivations to engage in health care depend on barriers and facilitators on 3 levels: individual, relational, and systemic. Similarly, our study results indicate that the motivations of patients with HF to co-produce health care are influenced by (1) individual factors (HL and self-efficacy), (2) relational factors (patients’ and professionals’ listening and communication skills), and (3) systemic factors (understanding of co-production, health care culture, and resource constraints).

### Individual and Relational Factors Influencing Co-Production

Mirroring previous research, our findings indicate that patients with HF have low levels of HL [31,56,57]. This includes insufficient skills to actively participate in everyday activities and apply new information to new circumstances as well as insufficient skills to analyze information and get greater control over life. Participating professionals suggested that patients’ willingness and capability to communicate with health care professionals might vary across generations. This represents a barrier to co-production—particularly among older persons—if it causes patients to refrain from communicating their own needs. Low self-efficacy, indicated here by patients’ assumption that their lived experiences would not be useful when designing care, has previously been acknowledged as a barrier to co-production [9].

Our findings suggest that professionals struggle to convey information to persons with low levels of HL, indicating that there is room for improvement in the organization’s HL (ie, professionals’ capabilities and communication skills as they relate to meeting the special needs of people with limited HL). Quality improvement efforts addressing HL issues and strengthening the professionals’ communication skills can increase organizational HL [58]. Palumbo and Manna [10] argued that limited organizational HL is a barrier to co-production when preventing the “evolution of health care providers from disease relievers—that is to say self-reliant and specialized healers of ill health status—to enablers—that is to say facilitators of patients’ activation and involvement in the provision of care.” Furthermore, professionals tend to overestimate patients’ HL levels, thus impairing patient-professional communication [59,60]. Although not explicitly expressed by the professionals in our study, an overestimation of patients’ HL levels could explain the struggle of professionals to convey health information to patients. Inviting family members to co-produce health and care might compensate for patients’ low levels of HL [61] and improve patients’ quality of life [62]. However, our findings mirror those from a study in the US Veterans’ Health Administration that suggest that family members want to participate in health and care decisions with and for patients with HF but feel excluded from care teams [63].

### Systemic Factors Influencing Co-Production

The study participants understood co-production to be the addition of something “new” on top of traditional health care. This mirrors the results of Alami et al [64], who found that citizen-patient involvement in shared decision making, a key component in co-production, was understood as a theorical idea rather than a current practice in health care. Even after explanation of co-production as an approach to improving health care processes on different organizational levels, it was mainly discussed regarding one-on-one interactions and rarely as an approach for improving health care on system levels. Gilardi et al [65] proposed that co-production can be seen from different perspectives: the first perspective focuses on patients and professionals interacting around clinical issues in the microsystem, and the second perspective focuses on multiple stakeholders from different organizations interacting on many organizational levels in different service delivery phases. Our findings indicate that the system perspective on co-production was not yet established among our respondents in chronic care settings.

**Figure 1:**
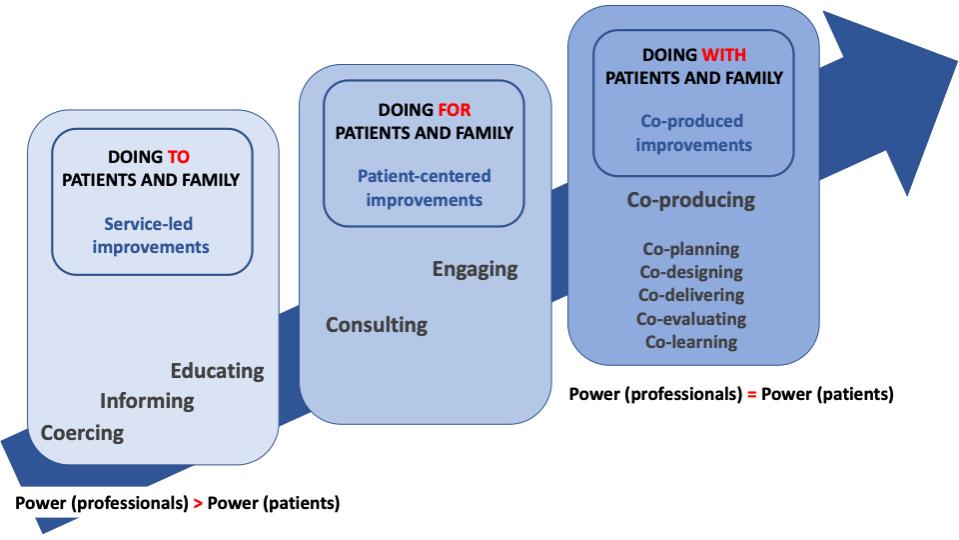
The co-production pathway (modified by lead researcher A-MS after Arnstein [66], Slay and Stephens [67], and Williams and Caley [68]).

Our Swedish study participants tell of a rather traditional “doctor-knows-best” health care context with professionals being in charge. As stated by some of the professionals in this study, old traditions, not having the time to invite patients and family members to co-produce health and care, and professionals’ fear of losing control over health care visits and processes may perpetuate the unequal balance of power between stakeholders [1,7-9,12,14]. Arnstein [66] proposed a “citizen ladder of participation” that drew attention to the power balance between stakeholders on a spectrum of participation, from manipulation to citizen control. Figure 1 shows a modified ladder, describing different levels of patient participation [66-68].

In our study, patient participation was described in terms of “informing” and “educating” patients and family members, sometimes moving over to “consulting” them (Figure 1). This implies that quality improvements in health and care in our study context are usually service-led rather than co-produced. Managing the barriers reported here—varying levels of understanding of the concept of co-production, limited individual and organizational HL, unease with power sharing between patients and professionals, and resource constraints—is key to moving toward a more equal balance of power between stakeholders in our study context.

### Methodological Considerations

This study was limited to one particular setting. The barriers and facilitators to co-production reported here may be different from those in other health care contexts. For example, HL can vary between patients suffering from chronic diseases other than HF. Also, stakeholder understanding of co-production of health and care and experience with involving patients and family members in care processes can vary across health care organizations, leading to preconditions other than those reported in this study. This limits the generalizability of our study findings. However, the findings from this study can be useful to reflect upon for other health care and chronic care settings when considering adopting co-production approaches.

Individuals who were unable to consent to participation and individuals without knowledge of the Swedish language were excluded from this study. The researchers recognize that this might have limited the selection of participants to individuals who are considered easy to co-produce health and care with, thus limiting the generalizability of the study findings. However, being able to participate in interviews in Swedish made it easy for the participants to share experiences in depth. Among patients and family members, there could have been a selection bias if only participants with positive experiences of health care had been invited and/or decided to participate. There were no indications that this was the case, as the participants shared both positive and negative experiences in the FGIs. Because of the similarities between the interview guides for different stakeholders, the guides were not tested with family members prior to the interview. There were no indications of family members not understanding the interview questions. Because of privacy concerns, patients’ and family members’ backgrounds, such as level of education and profession, were not mapped. This may hamper the ability to generalize the study findings.

The lead researcher (A-MS) works as a cardiologist in the study context. Thus, there might be a risk of bias due to the researcher’s close relationships with the professionals. In particular, there may be a risk of “social desirability” to influence their responses [69]. While good contextual knowledge is valuable when interpreting data, there is a risk that the lead researcher’s deep preunderstanding of this context could have made her unaware of some perspectives. Data analyses were reviewed with senior researchers (all coauthors)—a form of investigator triangulation—to strengthen the study’s trustworthiness.

### Conclusions

Co-production can be facilitated by stakeholders’ motivations. However, varying levels of understanding of co-production, limited HL, unease with power sharing between patients and professionals, and resource constraints are barriers that need to be managed to promote co-produced care and better health for persons living with HF. Further research is warranted to explore how to co-produce health care services with patients with HF in ways that are resource efficient and how leaders can facilitate the inevitable cultural change it requires and represents.

## Abbreviations

COM-B: Capability, Opportunity, and Motivation Behavior
FGI: focus group interview
HF: heart failure
HL: health literacy
PCC: primary care center
RJC: Region Jönköping County
